# Pediatric Humeral Shaft Fracture Nonunion: A Case Report

**DOI:** 10.7759/cureus.51019

**Published:** 2023-12-24

**Authors:** Matthew Lipphardt, Mazen Zamzam, Ehab S Saleh

**Affiliations:** 1 Orthopaedic Surgery, Beaumont Health, Royal Oak, USA; 2 Orthopaedic Surgery, Oakland University William Beaumont School of Medicine, Auburn Hills, USA

**Keywords:** pediatric trauma, nonunion fracture, humeral malunion, pediatric fractures, humeral shaft fracture

## Abstract

Humeral shaft fractures in the pediatric population are a commonly encountered injury in everyday practice. Most patients with these injuries are treated without surgery and go on to have an uneventful recovery. Nonunion of these injuries in the pediatric population has been reported only once in the literature. This case report follows a 13-year-old female after a seemingly standard transverse humeral shaft fracture. The patient was treated with a fracture brace initially. No signs of healing were noted at the eight-week post-injury follow-up. The family elected for continued conservative management until the patient returned at four months post-injury with persistent gross motion at the fracture site and no healing on radiographs. Laboratory testing did show that she has mild-to-moderate vitamin D deficiency, which was addressed. The patient underwent nonunion treatment with open reduction, internal fixation, and bone grafting. She went on to full union with an uncomplicated postoperative course. This case presents an interesting and unique case presentation. This report shows that, while rare, it is a potential outcome of humeral shaft fractures in the pediatric population. This case also demonstrates that using the standard adult operative technique for nonunion treatment with rigid internal fixation and bone grafting in a pediatric patient will lead to full-bone healing.

## Introduction

Pediatric humeral shaft fractures are common orthopedic injuries. Among the pediatric population, nearly all humeral shaft fractures are treated non-operatively [1]. Operative intervention is rare and is typically reserved for cases with an additional variable such as ipsilateral forearm injury, multiple injured patients, and associated nerve injury [1]. Non-operative management is the primary mode of treatment. This is due to the high tolerance for non-anatomic alignment of the humerus shaft as well as a large remodeling and healing potential in this population [1].

Multiple large studies evaluating the outcomes of pediatric humeral shaft fractures report that all patients went on to union. Due to this, there is no reported rate of pediatric humeral shaft fracture non-unions. There is also no consensus on the timeframe for giving the diagnosis of a nonunion in the pediatric population. Some studies report that a nonunion can be diagnosed in the pediatric population between 11 and 15 weeks from injury [2].

In contrast, the adult population has a well-established definition of nonunion. An adult humeral shaft fracture is diagnosed as a nonunion if there is no healing at nine months, or if there is no sign of progressive healing on subsequent X-rays three months after injury. This provides a comparison for the pediatric population who will typically heal fractures faster than their adult counterparts.

## Case presentation

**Figure 1:**
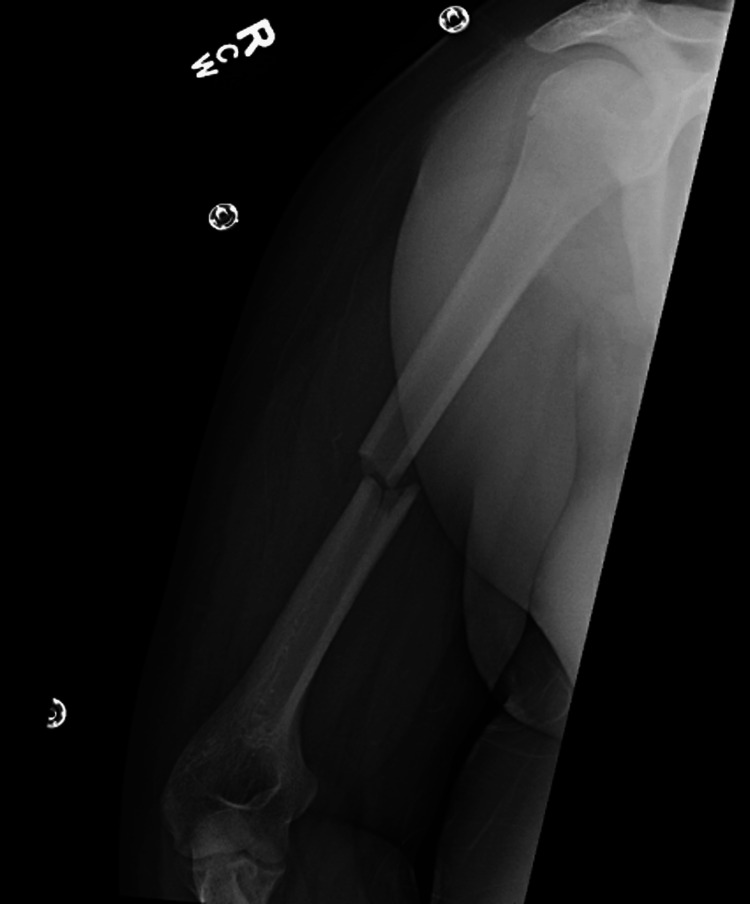
AP radiograph of the right humerus taken on the day of the injury in the emergency room. The image shows a transverse fracture of the middle humeral shaft with minimal comminution.

A 13-year-old female presented to our hospital with a chief complaint of traumatic right arm pain. She reported that she tripped while dismounting an ATV and fell directly onto her right shoulder. The patient was found to have a minimally displaced transverse humeral shaft fracture in the central third of the shaft (Figure 1). The patient was found to not have sustained any neuro-vascular injuries upon physical examination. In the emergency room, she was placed in a coaptation splint and sent home with a plan to follow up. She followed up one week after the injury and was transitioned to a custom-fitted Sarmiento brace with no motion allowed.

**Figure 2:**
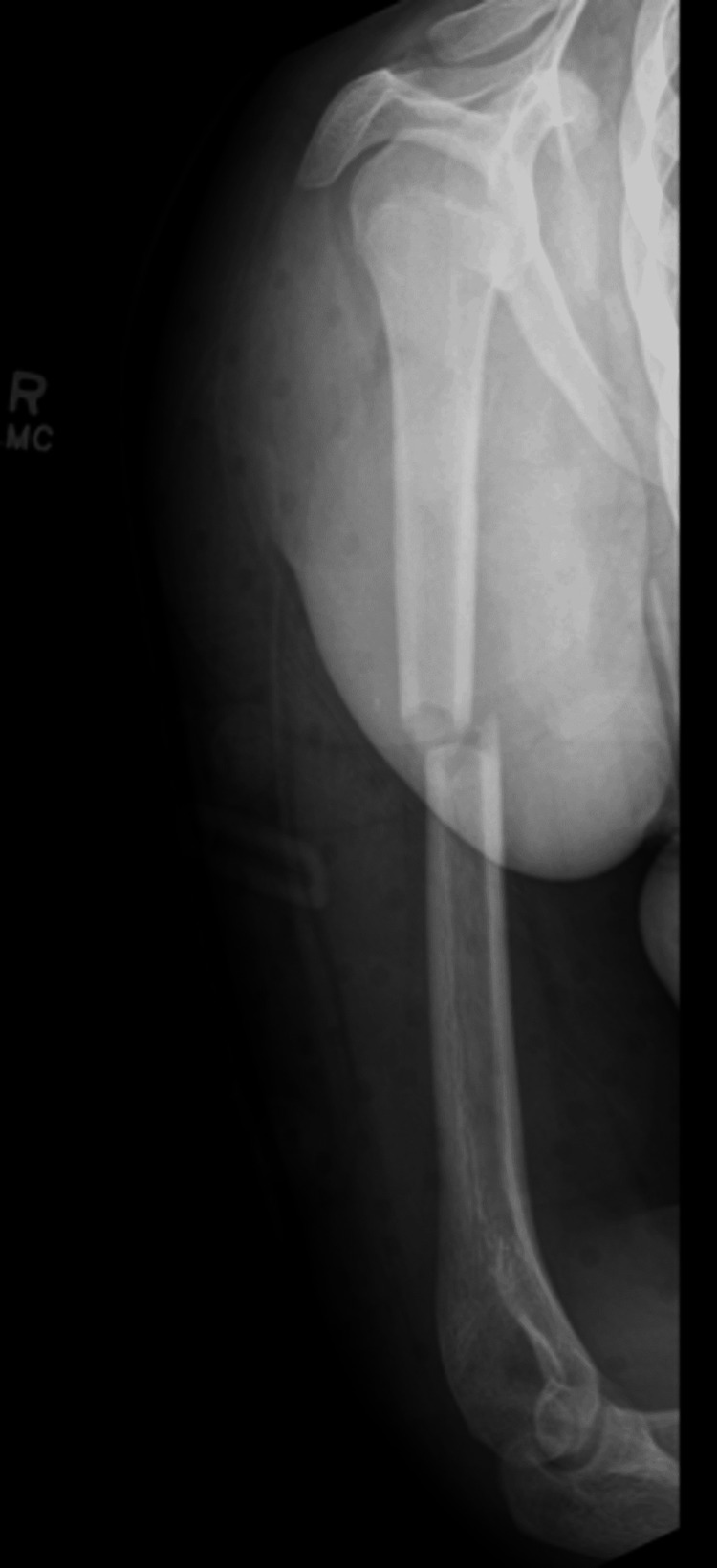
Lateral radiograph of the right humerus at six weeks post-injury. Redemonstration of transverse humeral shaft fracture. No callus formation noted. Fracture appears to maintain good alignment.

The patient reported for follow-up at six weeks post-injury for repeat radiographs, and there were minimal signs of healing. Labs were ordered to rule out infection and vitamin D deficiency. She was diagnosed with mild-to-moderate vitamin D deficiency, and her inflammatory markers were normal. The patient had continued restrictions on weight bearing and physical activity, and we recommended that she continue to use the fracture brace for six more weeks and initiate treatment for her vitamin D deficiency with 50,000 IU of vitamin D2 every week for eight weeks. At this time, the fracture demonstrated maintained alignment without any appreciable callus formation (Figure 2).

**Figure 3:**
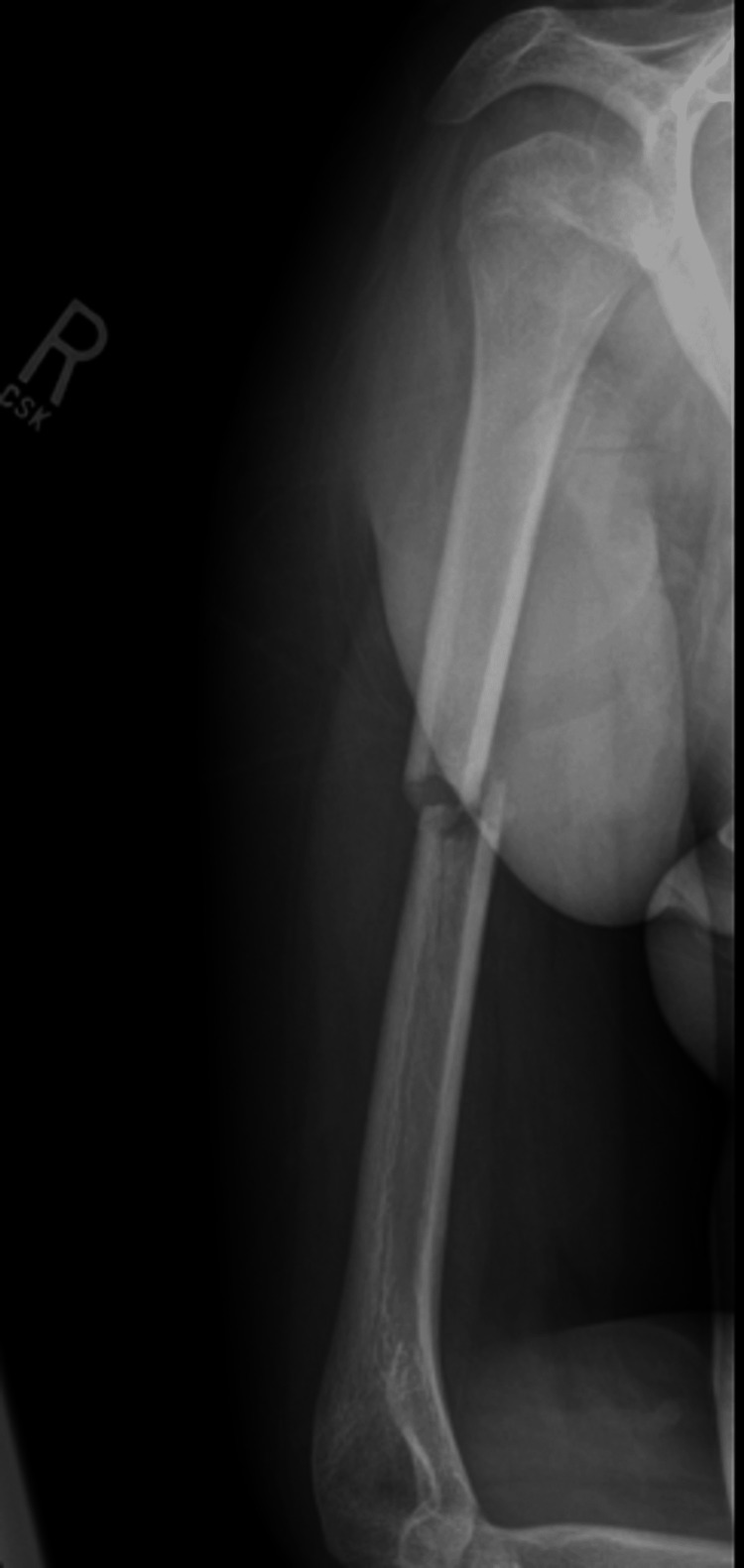
Radiograph of the humerus at 11 weeks post-injury. No callus formation noted. No bone overgrowth or hypertrophy of the fracture site noted.

At approximately 11 weeks post-injury, the patient reported continued pain in her arm with shoulder motion. There was gross motion and tenderness of the midshaft humerus fracture site and limited shoulder motion on the exam. Imaging at this time again showed maintained alignment, but minimal signs of callus formation were noted (Figure 3). At this time, he was diagnosed with delayed union. This was discussed with the patient and her parents, and treatment options were discussed, which included internal fixation with flexible nails vs continued treatment with the fracture brace for six more weeks, and a bone stimulator. The bone stimulator was prescribed with the intention of encouraging bone growth via electric current and shockwave therapy. The family elected to continue treatment with the fracture brace, and the newly prescribed bone stimulator.

**Figure 4:**
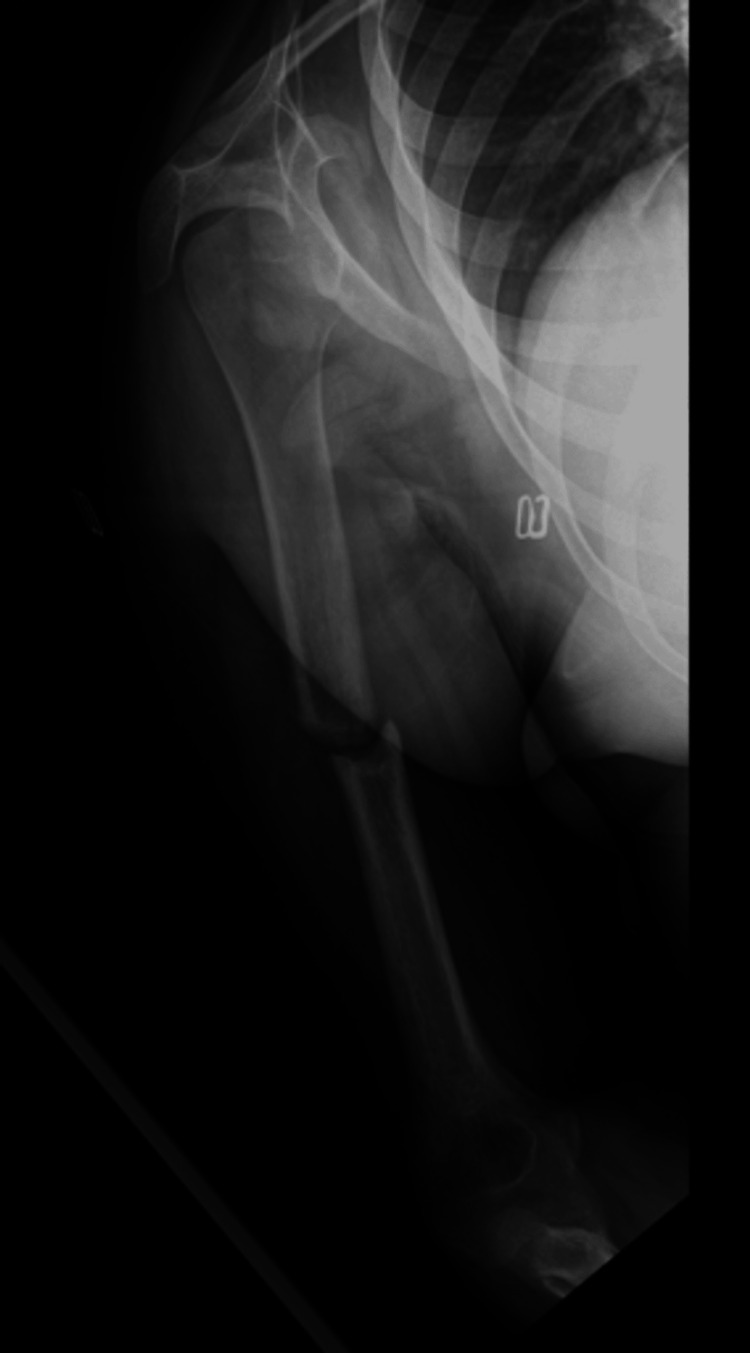
Radiograph of the humerus at five months post-injury. It demonstrates transverse humeral shaft fracture nonunion. No bone formation at the fracture site and no signs of progressive bony changes compared to prior images.

At a follow-up appointment 17 weeks after the injury, the patient continued to have gross motion of the fracture site and pain with activity. Given the persistent symptoms and radiographic findings (Figure 4), we recommended treatment with open reduction and internal fixation using compression plates and screws and bone grafting as we felt that flexible nailing at this point will have a high chance of failure. The patient has not been using the prescribed bone stimulator at this point, due to insurance-related reasons. She was referred to pediatric endocrinology to rule out other causes that could contribute to the nonunion and due to her obesity (BMI: 38.1), but no additional diagnosis was made. The family did not want to proceed with surgery at that point.

At 5.5 months post-injury, follow-up surgical treatment was decided. At 5.5 months post-injury, the patient was brought to the operating room for treatment. Under general anesthesia, the patient was positioned supine with the right upper extremity on a hand table. The anterior approach to the humeral shaft was used. The incision extended to the delto-pectoral interval proximally and the biceps-brachioradialis interval distally. The deep fascia was incised and the biceps muscle was retracted medially,The brachialis muscle was split to provide access to the humeral shaft fracture site. There was a pseudocapsule of fibrous tissue at the fracture nonunion site. Debridement of the capsule revealed the fracture ends with interposed soft tissue and persistent distraction of the two ends.

**Figure 5:**
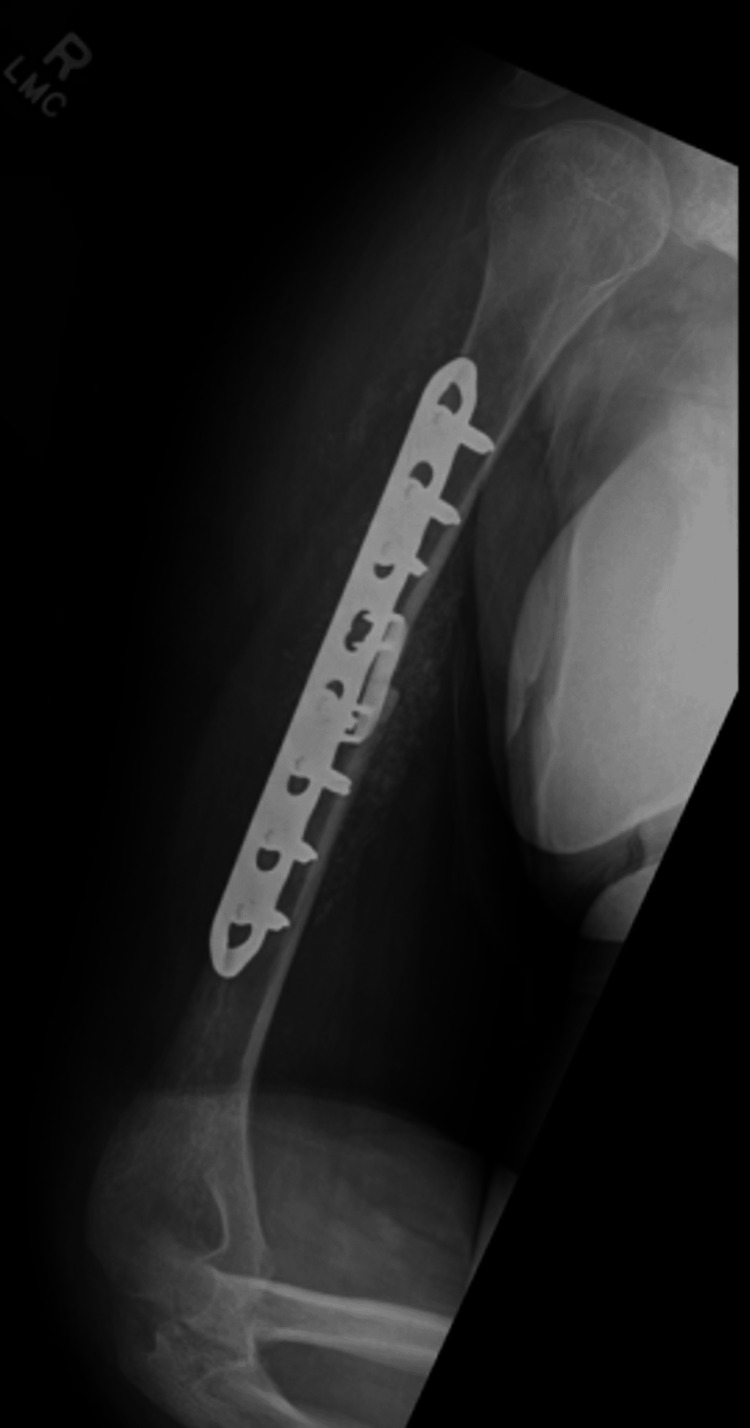
Immediate post-operative radiograph of the right humerus. Eight-hole plate and nitinol staple noted bridging the fracture site.

The medullary canal was opened on both ends. The fracture ends were taken back -2mm with an oscillating saw. Bleeding bone edges were produced. A Synthes BME Elite continuous compression implant, 4-legged, size 30 x 20 mm compressive staple was applied and produced compression at the fracture site. This was followed by a Synthes large fragment locking compression 8-hole 4.5 mm plate. Cellular bone graft (ViviGen), 15 cc was applied, and the surgical site closed. A postoperative radiograph is shown in Figure 5.

The postoperative course included a shoulder immobilizer for the first two weeks. By this time, the bone stimulator was approved by insurance, and the patient started using it for 30 minutes every day. A home range of motion exercise program was prescribed for the shoulder, elbow, and hand. The patient returned for a second postoperative visit at week four. X-rays at this time demonstrated earlier callus formation. The patient reported excellent pain control. The patient was limited from weight bearing and physical activity until week six.

**Figure 6:**
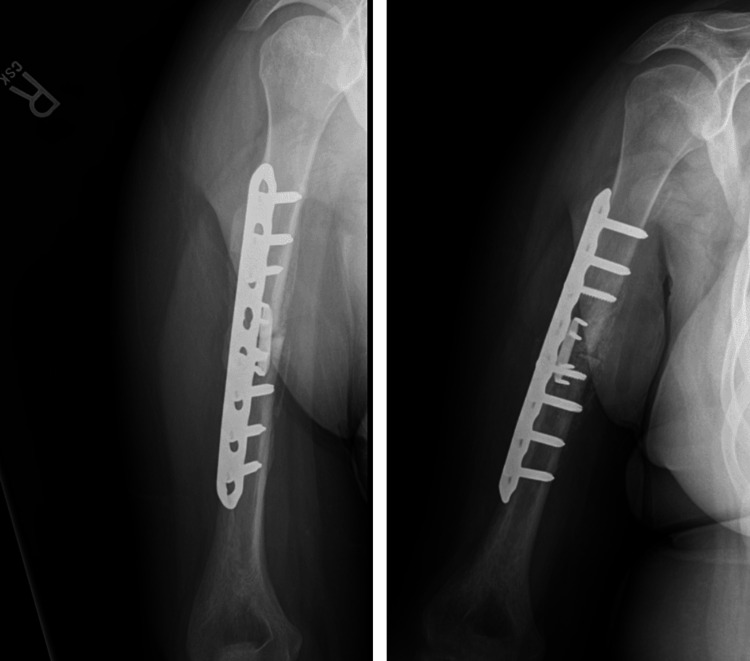
X-rays of the right humerus at a three-month post-operative visit. A healing reaction surrounding the bone is noted extending proximal and distal to the fracture site.

At three months postoperatively, the patient continued to do well. X-rays at this time showed robust callus formation and continued signs of healing (Figure 6). The patient had regained a full range of motion of the shoulder and elbow.

**Figure 7:**
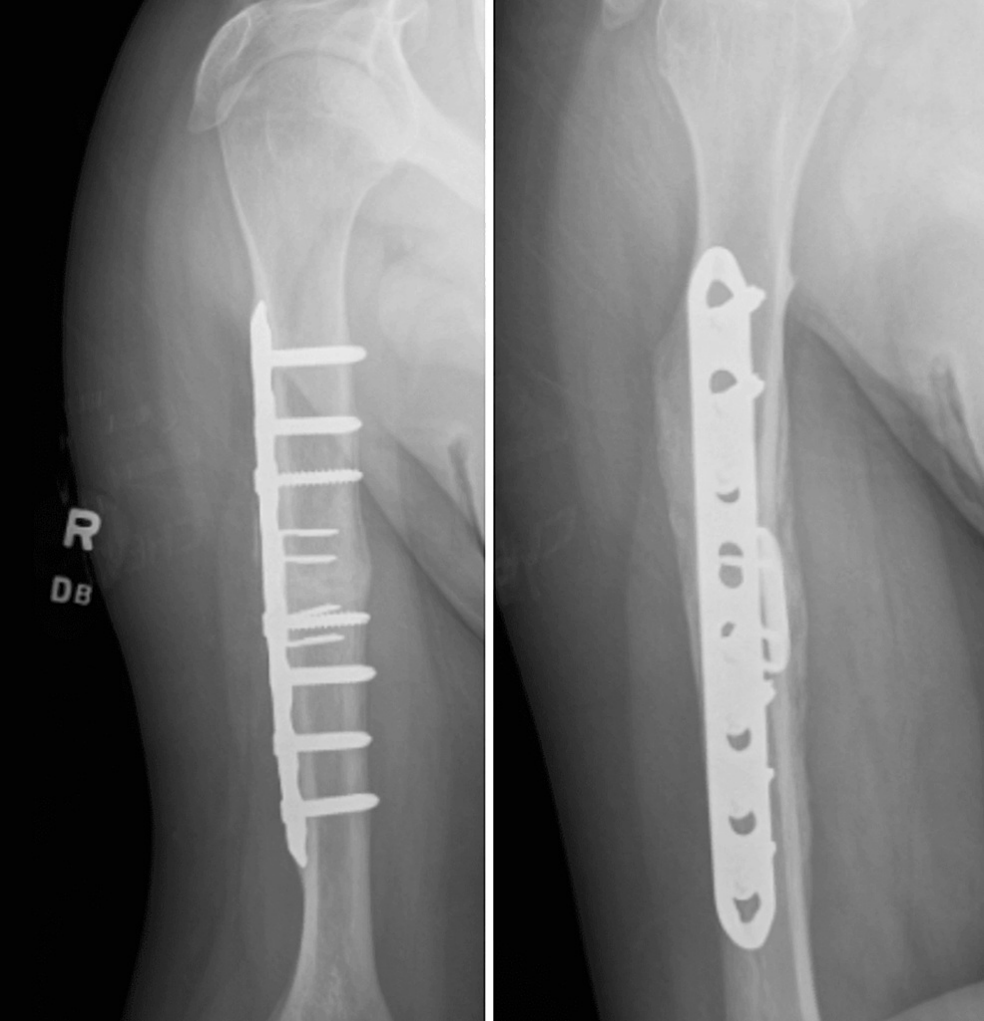
X-rays of the right humerus at eight months post-op. Complete healing of the fracture site is demonstrated.

The final postoperative visit was at eight months post-surgery (Figure 7). The patient reported no pain in the arm. Full strength and range of motion had been achieved in the shoulder, elbow, and hand. X-rays at this time showed complete union of the fracture site on all views with no signs of hardware loosening or lucency. The patient was advised to continue their activities as tolerated with no medical restrictions.

## Discussion

Adult humeral shaft fractures are relatively common, constituting 5-8% of all fractures within the adult population. Of those fractures, nonunion is found to be present in 15% of cases [3]. The majority are attributed to fragility-based fractures [3,4]. Nonunion is often described as having a bi-modal distribution. The majority of cases consist of atrophic fall fractures in patients 65 years and older. Within patients under 45 years old, nonunion is due to a high-energy trauma [4].

In the pediatric population, humeral shaft fractures are often attributed to accidental, low-energy traumatic force [5]. Fractures of the humeral shaft constitute 2-5% of all fractures in children [5]. Surgery is rarely indicated for acute pediatric humerus shaft fractures. They are typically treated with initial reduction and splinting, followed by a fracture brace to allow the fracture to heal [5]. Indications for surgery typically include open fracture, polytrauma, and loss of acceptable alignment [3]. Nonunion, in general, is a rare occurrence in children, due to the excellent bone healing and bone remodeling demonstrated in children [6]. Coupled with the low incidence of humeral shaft fractures within the pediatric population, it is not surprising that pediatric humeral shaft nonunion is an extremely rare complication. 

There are several factors that may increase the risk for nonunion in pediatrics, such as metabolic deficiency, and diabetes. Diabetes can increase osteoclast formation in fracture healing, which in turn increases bone resorption [7]. This is due to the reduced formation and function of osteoblast cells, as well as the number of osteoblasts readily available. This can cause nonunion within a fracture due to the reduced ability to create quality bone formation. The leading two causes of nonunion of a fracture within pediatrics are vitamin deficiencies and underlying pathologies of bone [8]. Osteoporosis patients are 2.75 times more likely to have a nonunion. This can be attributed to reduced bone mass and increased fragility found within osteoporotic patients. The second leading cause could be a vitamin D deficiency, which has been shown to put patients at a 2.91 times increased risk for nonunion in pediatrics [8]. Other conditions, which have also been shown to have an effect on fracture healing, are obesity, smoking, hypertension, antibiotic treatment, prescription opioid use, and bisphosphonate use [8].

This patient had two risk factors for nonunion, obesity and nutritional vitamin D deficiency [9]. Within the majority of nonunion cases, an underlying condition or deficiency can be identified and labeled as a predisposing cause. Within this case, obesity and a vitamin D deficiency could have been attributed to the potential nonunion of the fracture within the patient. An increased BMI and a decreased vitamin D level can severely impact osteogenesis and bone modeling processes, which can only worsen healing times [8].

Depending on the different classifications of nonunion, treatment can differ. Operative strategies differ based on whether or not vascularization is present. Treatments can include a fixed-angle locking plate, bone allografts, intramedullary nails, an external fixator, and a combination of previous methods [6]. In addition to allografts being used, synthetic and autografts can also be used as well. There are many factors that can indicate the usage of one graft in comparison to another. In the case of allografts and autografts, they can be used due to the increased osteoconductive and osteoinductive properties and support, as well as decreasing immune rejection [10]. For autografts, however, there is typically another incision done in order to harvest the graft. This can increase the risk of donor site complications, as well as limited availability of the graft. Synthetic grafts are often made via polymers and ceramics and have very little osteoconductive and osteoinductive effects. They mainly provide stability and structure and serve the purpose of providing a scaffold for new bone growth as well as into surrounding tissue [10].

In this case, the nonunion can be described as an atrophic nonunion. There are few cases regarding humeral shaft fracture nonunion within the adult literature. In 2016, Miska et al. [8] conducted a retrospective chart review, looking at the incidence of humeral shaft fracture nonunions. A limitation of this article was that inclusion criteria clearly stated that patients must be over 18 years of age; however, one patient included within the dataset was a 14-year-old patient.

The patient described in the study was a 14-year-old child who presented with a humeral shaft fracture nonunion. The patient had a minimal risk score and had not undergone any previous surgeries. The patient underwent surgery two months after the initial injury, using an allograft and fixed plate, and only took one month for the nonunion to completely heal. The article did not mention any risk factors that that the patient had and did not highlight any causes that could have led to nonunion within the patient.

## Conclusions

We present a rare pediatric occurrence of a humeral shaft nonunion, which can compromise shoulder and elbow motion, chronic pain, and low quality of life. At the same time, however, it can have excellent results once treated appropriately. Most pediatric humeral shaft fractures are treated conservatively with a fracture brace, with expected good results. However, nonunion of a pediatric humerus shaft fracture is an indication for operative treatment with rigid internal fixation and bone grafting.

Fixing pediatric humeral shaft nonunions with a compressional plate and screws with bone allograft usually gives good stability, with no reported displacement of fractures.
